# Supplementary material for: Pulsed laser induced plasma and thermal effects on molybdenum carbide for dry reforming of methane
Source: Nat Commun. 2024 Jun 28;15:5495. doi: 10.1038/s41467-024-49771-3 (PMC11214624; doi:10.1038/s41467-024-49771-3)
Supplement: Supplementary file 1 — Supporting Information [file 41467_2024_49771_MOESM1_ESM.pdf]

Pulsed Laser Induced Plasma and Thermal Effects on Molybdenum Carbide for Dry Reforming of Methane

Yue Li[1, †], Xingwu Liu[2, †], Tong Wu[1], Xiangzhou Zhang[1], Hecheng Han[3], Xiaoyu Liu[4], Yuke Chen[1], Zhenfei Tang[1], Zhen Liu[1], Yuhai Zhang[1], Hong Liu[1,4], Lili Zhao[1*], Ding Ma[2*], Weijia Zhou[1*]

1 Institute for Advanced Interdisciplinary Research (iAIR), School of Chemistry and Chemical Engineering, University of Jinan, Jinan, China

2 Beijing National Laboratory for Molecular Sciences, College of Chemistry and Molecular Engineering, Peking University, Beijing, China

3 Shandong Technology Center of Nanodevices and Integration, School of Integrated Circuit, Shandong University, China

4 State Key Laboratory of Crystal Materials, Shandong University, China

† These authors contributed equally to this work.

* Corresponding authors. E-mail: ifc_zhaoll@ujn.edu.cn (L. Zhao), dma@pku.edu.cn (M. Ding), ifc_zhouwj@ujn.edu.cn (W. Zhou)

Table of Contents

Supplementary Methods

Supplementary Figures

Supplementary Tables

Supplementary References

Supplementary Methods

Chemicals

Molybdenum trioxide (MoO_3) and barium sulfate ($BaSO_4$) were purchased from Shanghai Macklin Biochemical Co., Ltd.. Deionized water was supplied with a Barnstead Nanopure Water System (Smart2Pure 6UV,18.2 MΩ cm). Carbon dioxide (CO_2, purity 99.99%), methane (CH_4, purity 99.99%), Argon (Ar, purity 99.99%) were obtained from Jinan De Yuan Gases Co. Ltd., Hydrogen (H_2) was supplied with a CEHL-500 Hydrogen generator from Beijing China Education AuLight Technology (CEAuLight) Co., Ltd..

The photothermal catalytic performance test in the closed batch system

By placing the Mo_2C NSs (20 mg) on quartz cotton at the bottom of the quartz reactor, the mixed gas of CO_2, CH_4 and Ar at a ratio of 47.5%:47.5%:5% was poured into the reactor for 15 mins to remove air in the reactor, then closed the reactor vent. The light intensity of a 300 W Xe lamp with a plano-convex lens is 3 W cm^{-2}. The post-reaction gases were analyzed using a GC to obtain the relative amounts of CO, H_2, CO_2, and CH_4.

Quantification of reaction yields, selectivity, conversion, and energy efficiency

The reaction yields were calculated based on the mass of the Mo_2C:

$$\text{Yield}\left(\text{mmol}\cdot\text{h}^{-1}\cdot\text{g}^{-1}\right)=\frac{|\Delta\, \text{p}(\%)|\cdot \text{V (mL)}\cdot 60\left(\text{min}\cdot\text{h}^{-1}\right)}{\text{t (min)}\cdot \text{m}_{\text{Mo}_2\text{C}}\text{ (g)}\cdot 22.4\left(\text{mmol}\cdot\text{mL}^{-1}\right)} \tag{1}$$

The selectivity is expressed as the ratio of H_2 to CO produced:

$$\text{H}_2/\text{CO (sealed system)}=\frac{\left|\Delta\, \text{p}_{\text{H}_2}\,(\%)\right|}{\left|\Delta\, \text{p}_{\text{CO}}\,(\%)\right|} \tag{2}$$

$$\text{H}_2/\text{CO (flow system)}=\frac{\text{F}_{\text{H}_2,\text{ out}}}{\text{F}_{\text{CO, out}}} \tag{3}$$

The conversion of reactant was calculated using:

$$\text{Conversion}=\frac{\text{F}_{\text{in}}\text{-}\text{F}_{\text{out}}}{\text{F}_{\text{in}}}\times 100\% \tag{4}$$

The total energy efficiency of laser-catalysis and thermocatalysis was calculated using:

$$\text{E}=\frac{r_{\text{CH}_4,\text{converted}}+r_{\text{CO}_2,\text{converted}}}{\text{P}_{\text{output}}} \tag{5}$$

The total energy efficiency of photocatalysis was calculated using:

$$E = \frac{r_{CH_4,converted} + r_{CO_2,converted}}{P_L} \tag{6}$$

The electricity cost of laser-catalysis, thermocatalysis, and photocatalysis was calculated using:

$$C = \frac{r_{CH_4,converted} + r_{CO_2,converted}}{P_E} \tag{7}$$

Δ p refers to the percentage change of a product in the reactor; F_{in} and F_{out} refer to the reaction gas flow rate of inlet and outlet, respectively; $r_{CH_4,converted}$ and $r_{CO_2,converted}$ refer to rate of conversion of CH_4 and CO_2 (mmol s^{-1}), respectively; P_{output} refers to the output power of the pulsed laser (16 W) and the fixed-bed reactor (496 W); P_L refers to the luminous power of the Hg–Xe lamp (60 W, output power of the 150 W Hg–Xe lamp); P_E refers to the electric power of the pulsed laser (1200 W), the fixed-bed reactor (730 W), and the Hg–Xe lamp (150 W).

COMSOL theory simulations

In addition to the experimental study, the laser heating of $Mo_2C/BaSO_4$ tablet and the laser induced plasma of the mixed gas were theoretically simulated. The calculations were carried out in COMSOL Multiphysics® and the physical models used in the simulation modeling process included: unipolar ion drift model, reduced drift-diffusion model, complex chemistry kinetic model. The specific parameters of the modeling process were as follows: laser output power 16 W, laser spot radius 50 μm, laser pulse period 20 kHz, pulse width 100 ns.

Unipolar ion drift model:

$$\nabla\cdot\left(-\mu_e\vec{E}n_n\right)=0 \tag{8}$$

Drift-diffusion equations:

$$\frac{\partial n_e}{\partial t}+\nabla\cdot(-\mu_e\vec{E}n_e-D_e\nabla n_e)= \alpha n_e\left|\mu_e\vec{E}\right|-\eta n_e\left|\mu_e\vec{E}\right|-k_{ep}n_e n_p \tag{9}$$

$$\frac{\partial n_e}{\partial t}+\nabla\cdot(\mu_p\vec{E}n_p-D_p\nabla n_p)= \alpha n_e\left|\mu_e\vec{E}\right|-k_{np}n_n n_p \tag{10}$$

$$\frac{\partial n_e}{\partial t}+\nabla\cdot(-\mu_n\vec{E}n_n-D_n\nabla n_n)= \eta n_e\left|\mu_e\vec{E}\right|-k_{np}n_n n_p \tag{11}$$

Electrostatic protection:

$$\vec{E}=-\nabla\emptyset \tag{12}$$

Material transportation:

$$\nabla^2\emptyset = -\frac{e(n_p - n_e - n_n)}{\varepsilon} \tag{13}$$

Supplementary Figures

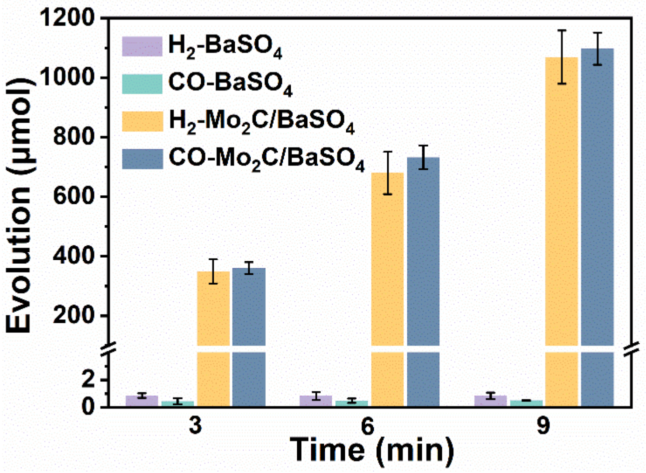

Supplementary Figure 1. Yields of H_2 and CO for $BaSO_4$ compared to that for $Mo_2C/BaSO_4$ via laser-catalysis. Performance evaluation was conducted in a closed system under focused irradiation with 16W pulsed laser. $BaSO_4$ tablets and $Mo_2C/BaSO_4$ tablets were used as catalysts to catalyze DRM in a mixed atmosphere for 3 min, 6 min and 9 min respectively. Error bars represent standard deviation.

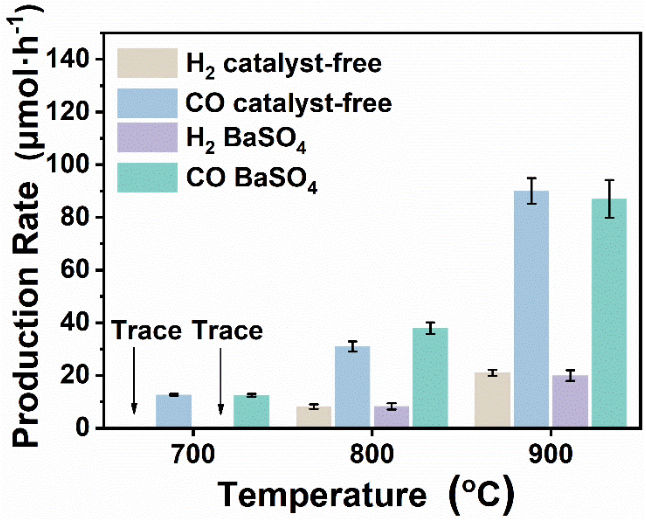

Supplementary Figure 2. The H_2 and CO production rates for $BaSO_4$ and catalyst-free via thermocatalysis. The thermocatalytic DRM of $BaSO_4$ powders in a flow system was carried out in a fixed bed quartz reactor at atmospheric pressure. The activity was evaluated at 700 °C, 800 °C, 900 °C and total flow rate of the feed gas (CO_2:CH_4:Ar = 16.7%:16.7%:66.6%, 20 mL min^{-1}). Error bars represent standard deviation.

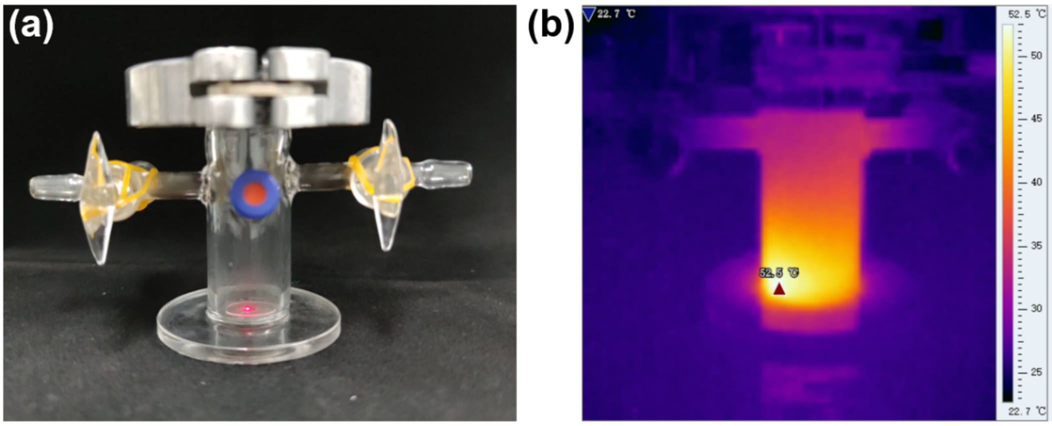

Supplementary Figure 3. (a) Photograph and (b) infrared thermography of the reactor for laser-catalytic DRM in closed system.

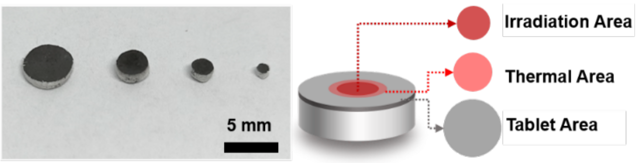

	Sample 1	Sample 2	Sample 3	Sample 4
Tablet Area (mm^2)	12.57	7.07	3.14	0.79
Thermal Area (mm^2)	0.95	0.95	0.95	0.79
Irradiation Area (mm^2)	0.69	0.69	0.69	0.69

Supplementary Figure 4. Schematic representation of various catalytic testing modes. Gray circle represents the catalyst tablet area, Light Red circle represents the thermal area of the catalyst tablets, Red circle represents the irradiation area of laser.

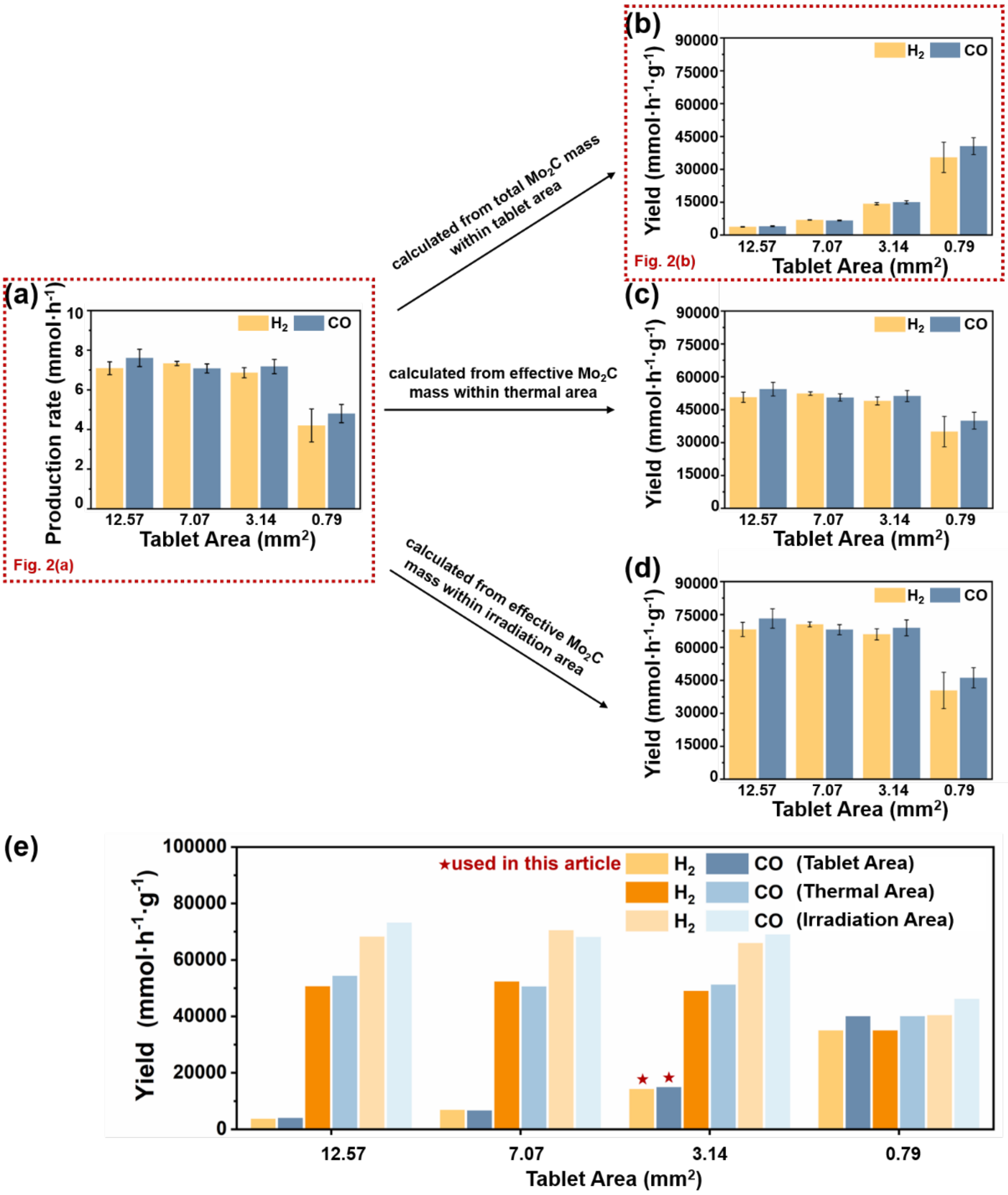

Supplementary Figure 5. Catalytic activity of the samples using the Mo_2C mass in three areas. Error bars represent standard deviation.

For the calculation of catalytic activity, we calculated the mass activity using the Mo_2C mass corresponding to these three areas respectively, and the results are shown in Supplementary Fig. 5 and Supplementary Table 1-3. The catalytic activity calculated by the total Mo_2C mass in tablet area is experimental activity, while the catalytic activities calculated by the effective Mo_2C masses in thermal area and irradiation area are theoretical activities. Obviously, the theoretical activity is much higher than the

experimental activity for Sample 1-3 with the tablet area of 12.57 mm^2, 7.07 mm^2 and 3.14 mm^2. In our experiment, the data we used was the experimental activity of sample 3 with tablet area of 3.14 mm^2, so we can understand that the activity of laser catalysis is underestimated.

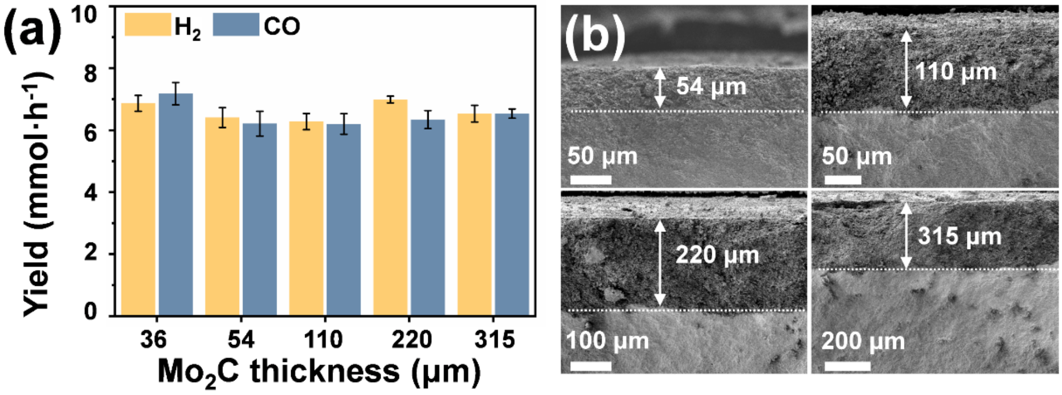

Supplementary Figure 6. (a) Laser-catalytic DRM performances and (b) SEM image of the cross-section of $Mo_2C/BaSO_4$ tablets with different thickness. Error bars represent standard deviation.

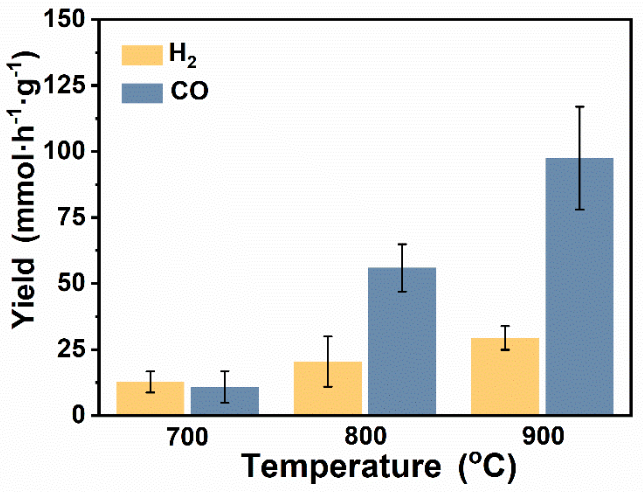

Supplementary Figure 7. The thermocatalytic DRM performances of $Mo_2C/BaSO_4$ tablet at different temperatures. Error bars represent standard deviation.

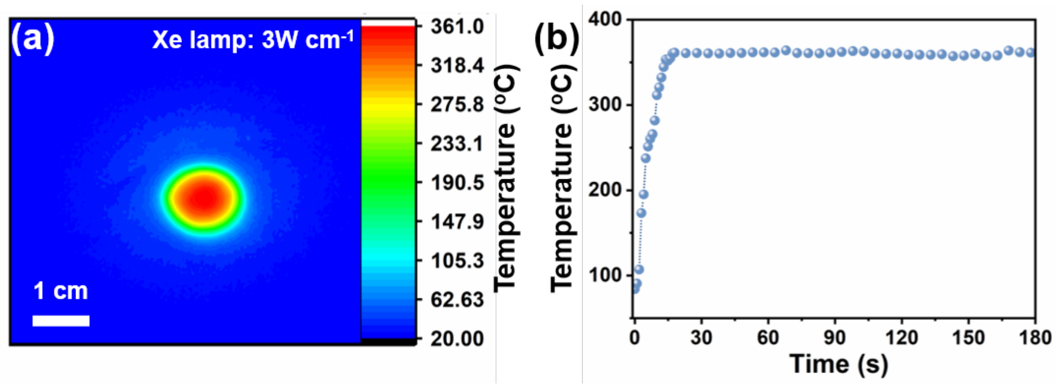

Supplementary Figure 8. (a) The infrared thermal imaging photo of Xenon lamp-driven photothermal catalytic DRM. (b) Temperature variation curve of Mo_2C with time under Xenon lamp irradiation

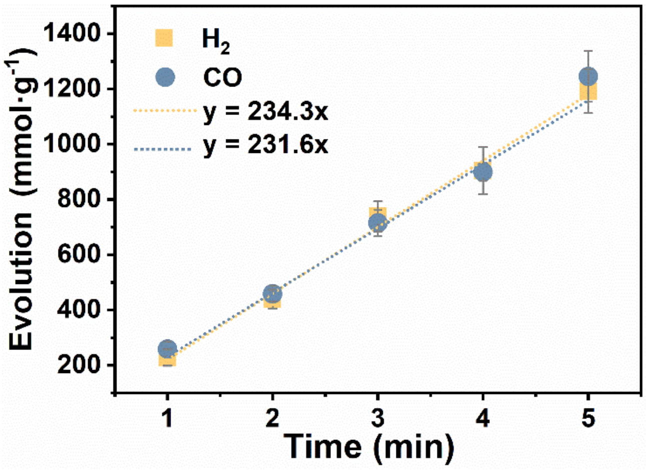

Supplementary Figure 9. Linear relationship of H_2 and CO production with time for $Mo_2C/BaSO_4$ tablet via pulsed laser-catalytic DRM. Error bars represent standard deviation.

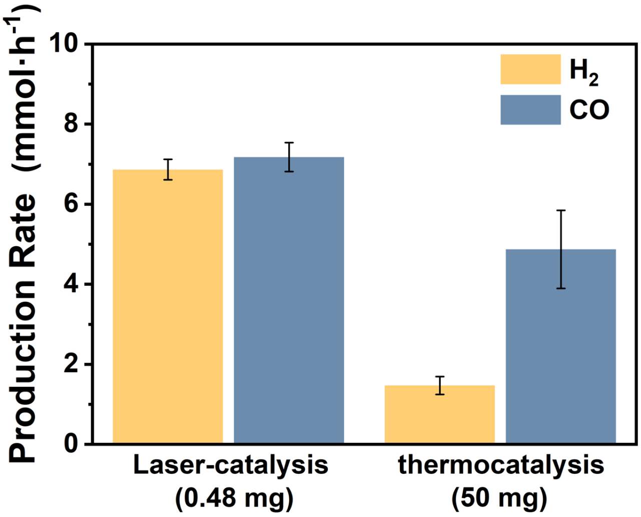

Supplementary Figure 10. The production rate of the laser-catalysis system with less catalyst (0.48 mg Mo_2C) and thermocatalysis system (50 mg Mo_2C). Error bars represent standard deviation.

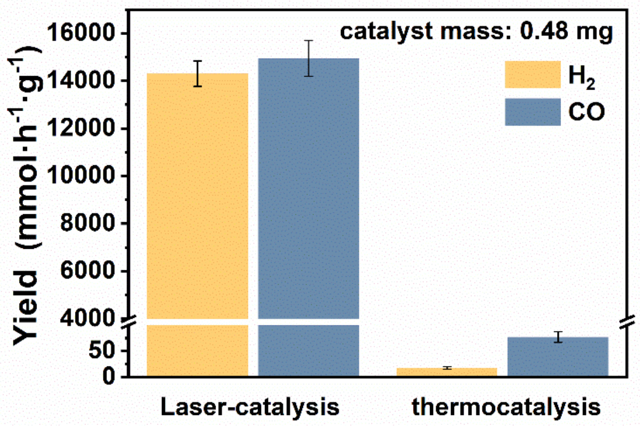

Supplementary Figure 11. Performances of the laser-catalysis system and thermocatalysis system with the same small amount of catalysts (0.48 mg Mo_2C). Error bars represent standard deviation.

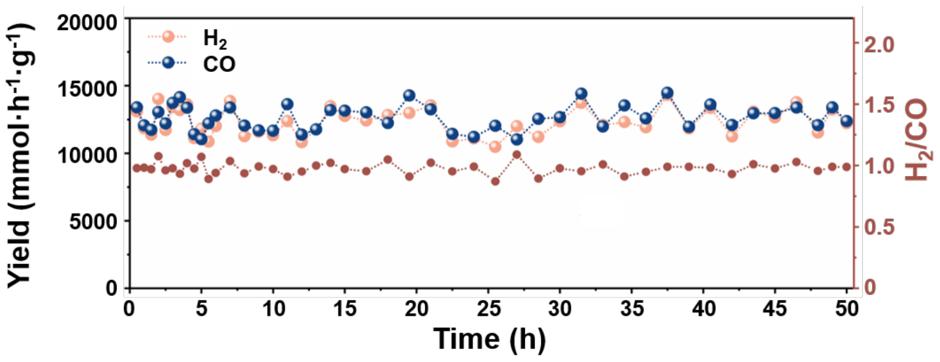

Supplementary Figure 12. Stability testing of laser-catalytic DRM using a $Mo_2C/BaSO_4$ tablet for 50 h (16W pulsed laser under infocused mode).

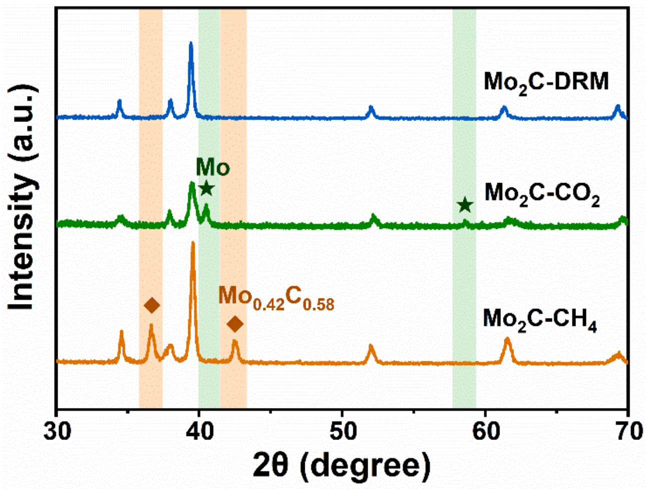

Supplementary Figure 13. XRD patterns of the $Mo_2C/BaSO_4$ tablet in laser-catalytic DRM reaction under CO_2 (CO_2： Ar = 95%:5%), CH_4 (CH_4： Ar = 95%:5%), and CO_2+CH_4 (CO_2:CH_4:Ar = 47.5%:47.5%:5%) atmosphere.

In pulsed laser-catalytic DRM, a C*-O* balanced environment created by the pulsed laser induced CH_4 cracking could avoid the deactivation of Mo_2C arising from deep oxidation by CO_2. Therefore, the equilibrium reaction between CO_2 and CH_4 was significant for pulsed laser-catalytic DRM catalytic stability. Using the CH_4 or CO_2 as reaction gas, the DRM catalytic reaction was carried out under C*-rich or C*-deficient atmosphere, respectively, which caused an irreversible phase change of Mo_2C catalyst. The XRD patterns of Mo_2C after laser-catalytic reaction in different reaction atmospheres revealed these irreversible reactions (Supplementary Fig. 13). Under pure

CO_2 atmosphere, two additional peaks at 40.52° and 58.61°, corresponding to (200), (220) crystal planes of Mo appeared. Under pure CH_4 atmosphere, the C* produced by methane cracking resulted in the formation of $Mo_{0.42}C_{0.58}$ with characteristic diffraction peaks at 37.07° and 42.83°. While under CO_2: CH_4 feed ratio of 1:1 atmosphere, Mo_2C phase remained intact, suggesting that the equilibrium reaction between CH_4 and CO_2 occurred. According to previous research, the O* produced by the reduction of CO_2 and the C* produced by the cracking of CH_4 further reacted to form CO under the catalysis of Mo_2C [1].

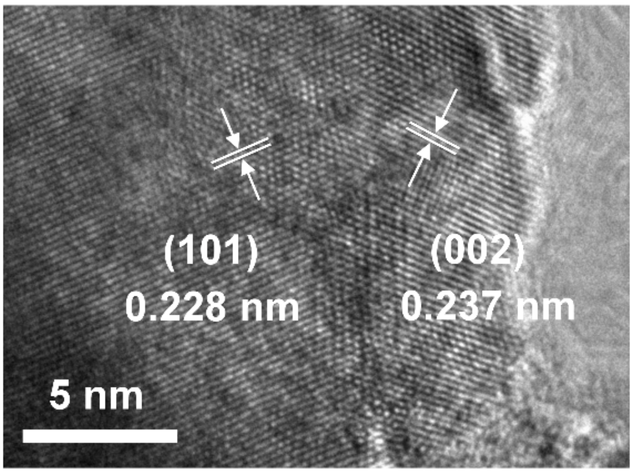

Supplementary Figure 14. HRTEM image of Mo_2C using FIB pick-up system from the laser irradiated areas.

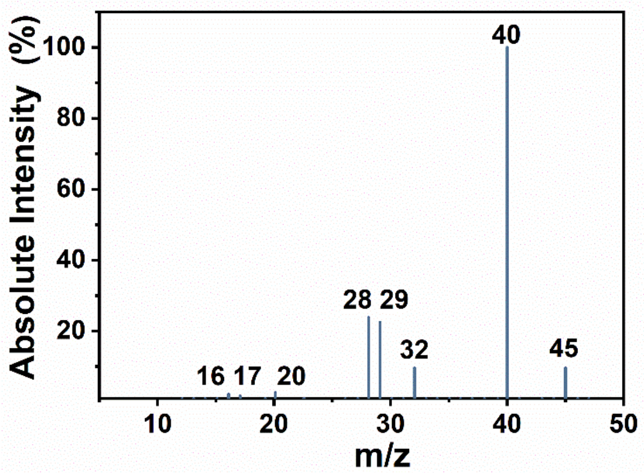

Supplementary Figure 15. Isotope-labeled $^{13}CO_2$ and $^{13}CH_4$ laser-catalytic tests screened by GC-MS. Mass spectrum of ^{13}CO and ^{12}CO (m/z 29, 28) produced over Mo_2C catalyst.

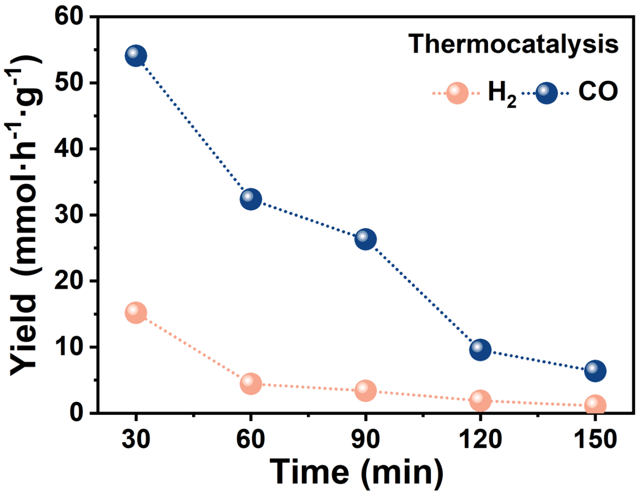

Supplementary Figure 16. Performance of the $Mo_2C/BaSO_4$ tablet in thermocatalytic DRM reaction at 800 °C over time.

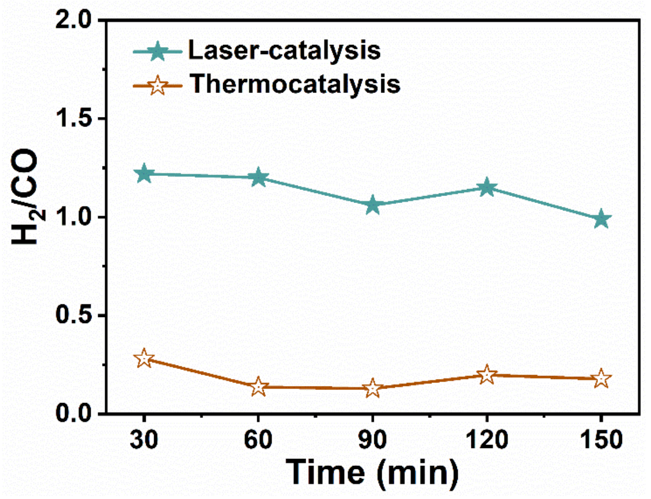

Supplementary Figure 17. Selectivities of laser-catalytic DRM in 16 W infocus mode and thermocatalytic DRM at 800 °C over time.

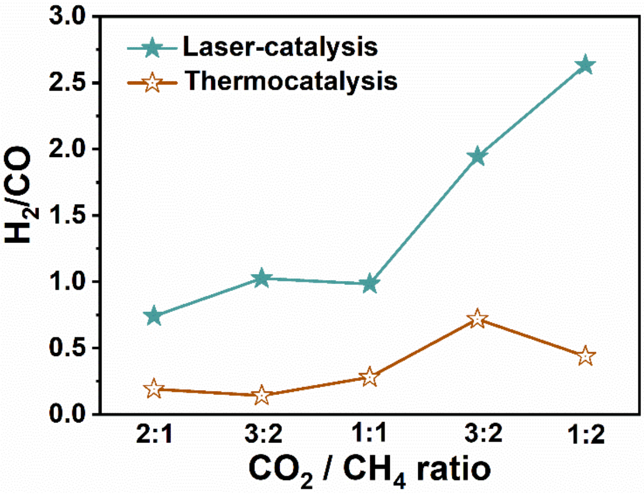

Supplementary Figure 18. Selectivities of laser-catalytic DRM and thermocatalytic DRM in different proportions of CO_2 and CH_4

The much lower selectivity with the H_2/CO molar ratio of 1:3.3 for thermocatalytic DRM compared with that for laser-catalytic DRM (Supplementary Fig. 17) also verified this conclusion. Even if the proportion of CH_4 in the reactants was increased, the product of thermocatalytic DRM was still dominated by CO. Conversely, the proportion of H_2 in the product increased with the proportion of CH_4 in the reactants in the laser-catalytic DRM process (Supplementary Fig. 18). It is well established that Mo_2C possessed ideal CO_2 activation capability, but was not dominant in methane activation [1, 2].

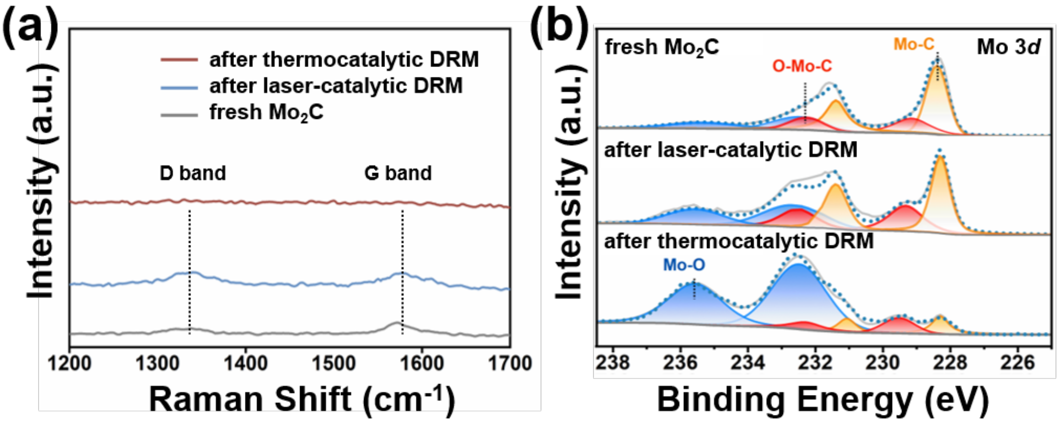

Supplementary Figure 19. (a) Raman spectra and (b) Mo 3*d* XPS spectra of Mo_2C before and after laser-catalytic DRM and thermocatalytic DRM.

As shown in Supplementary Fig. 19a, Raman pattern of the spent Mo_2C catalysts after DRM showed that fresh Mo_2C itself contains carbon peaks at 1578 cm^{-1} (G band)

and 1345 cm^{-1} (D band). After thermocatalytic DRM reaction, the D peak and G peak disappear, indicating that a C-deficient environment and the anti-coking advantages of Mo_2C. This resulted in C consumption and oxidation of Mo_2C. While in the laser-catalytic DRM, C-O equilibrium environment leads to neither carbon deposition nor oxidation of Mo_2C.

The XPS result also confirmed the structure stability of Mo_2C during pulsed laser-catalytic DRM process under CO_2:CH_4 feed ratio of 1:1 atmosphere, as shown in Supplementary Fig. 19b. Compared with the fresh catalysts, the intensity of the Mo-C peaks for Mo_2C were relatively invariable after laser-catalytic DRM. On the contrary, the intensities of the Mo-O peaks in Mo_2C after thermocatalytic DRM were significantly higher than that of the Mo-C peak, indicating the serious oxidation of Mo_2C, which caused the poor catalytic stability.

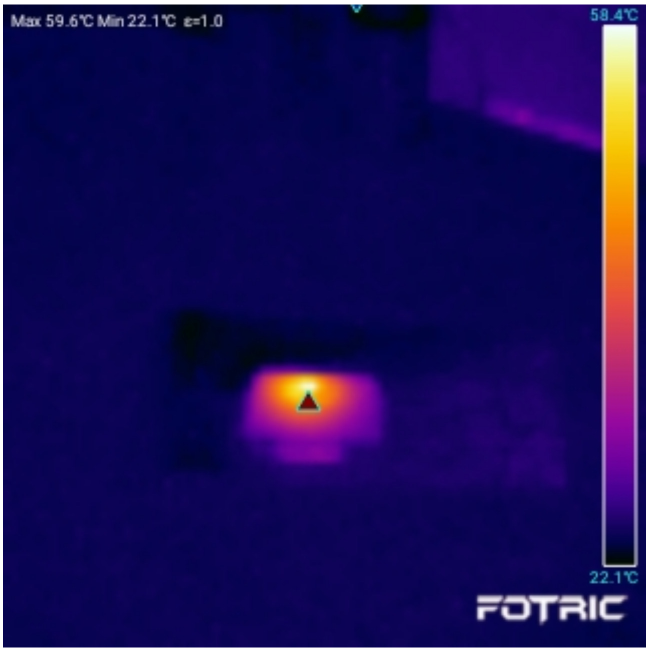

Supplementary Figure 20. Infrared thermal imaging photo of laser irradiating barium sulfate.

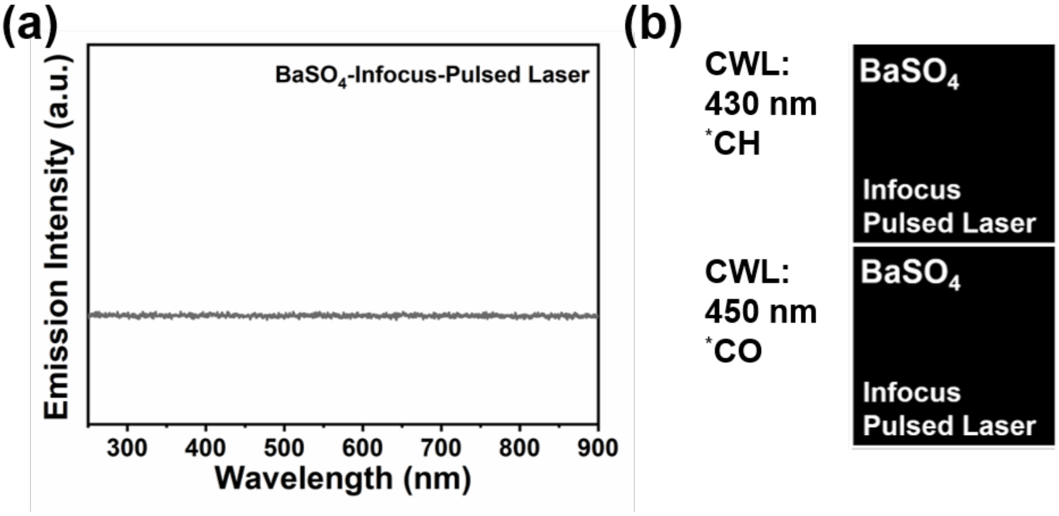

Supplementary Figure 21. (a) Excitation spectra and (b) the plasma images of laser-catalytic DRM under $BaSO_4$-Infocus-Pulsed Laser.

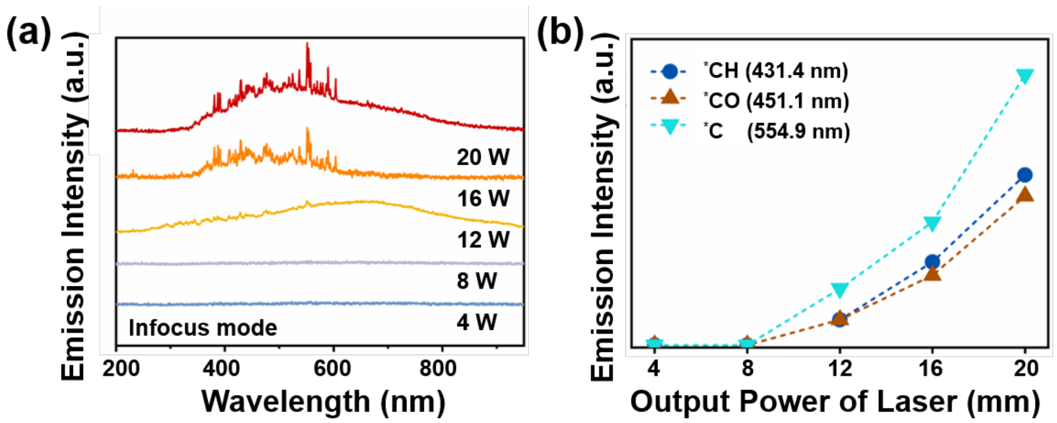

Supplementary Figure 22. Excitation spectra of laser-catalytic DRM for $Mo_2C/BaSO_4$ tablet at different output powers of pulsed laser in infocused mode.

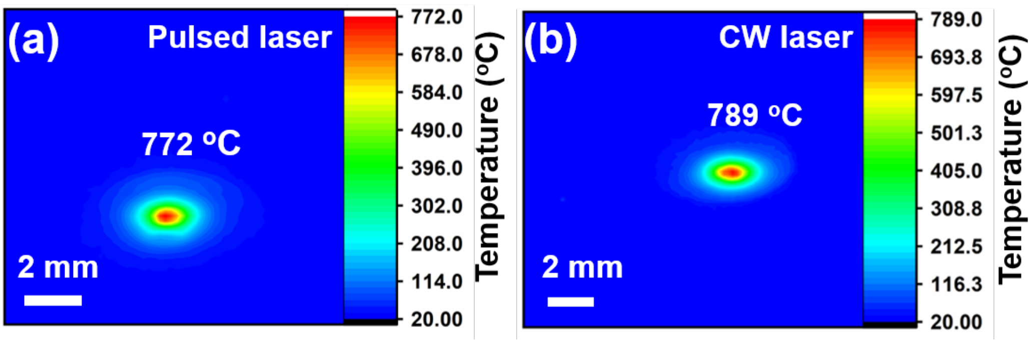

Supplementary Figure 23. The temperature generated by (a) 16W pulse laser focused irradiation and (b) CW laser irradiation on the $Mo_2C/BaSO_4$ tablet.

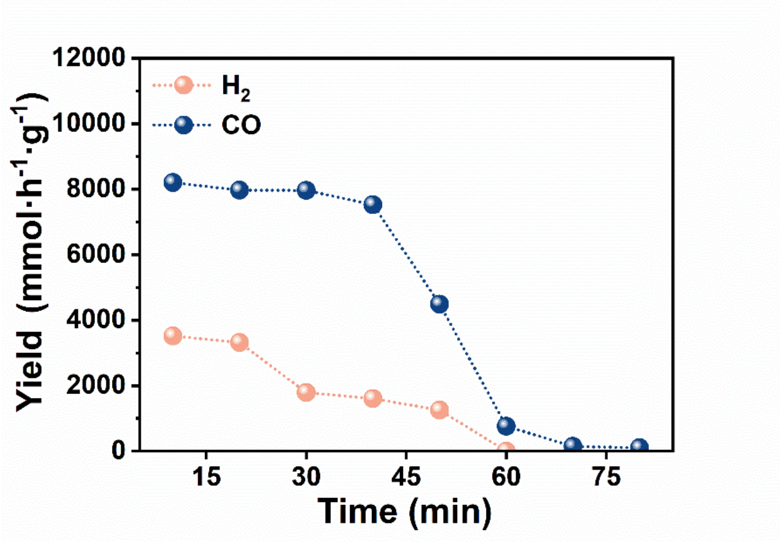

Supplementary Figure 24. The catalytic stability of Mo_2C in CW laser-catalytic DRM.

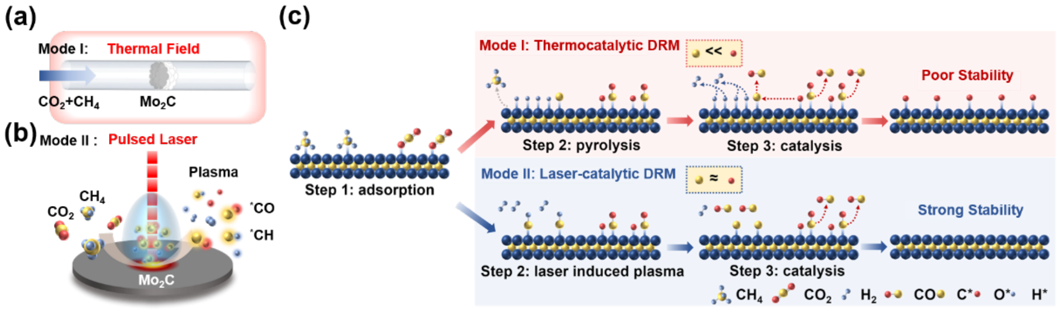

Supplementary Figure 25. Schematic diagram of (a) thermocatalytic DRM and (b) laser-catalytic DRM on Mo_2C and the corresponding reaction mechanism for these two strategies (c).

As the first step, the two CH_4 and two CO_2 molecules were adsorbed on the surface of the Mo_2C, which made no difference for thermocatalytic DRM and laser-catalytic DRM. As the second step, for the thermocatalytic DRM, one CH_4 molecule and two CO_2 molecules were cleaved to 4 H*/1 C* and 2 CO*/2 O* on Mo_2C via the thermocatalysis, and the unbalanced cracking reaction was attributed to the weak CH_4 cracking capacity of Mo_2C.

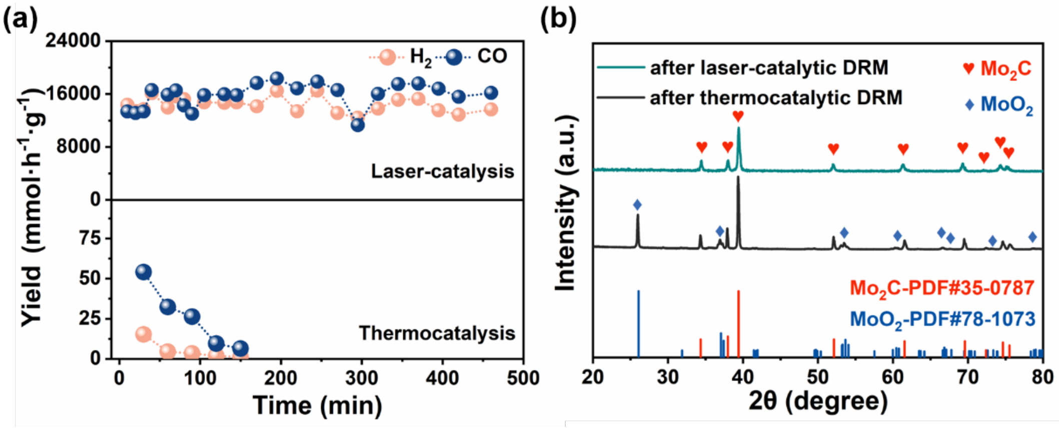

Supplementary Figure 26. (a) Catalytic stability and (b) XRD pattern of the $Mo_2C/BaSO_4$ tablet in laser-catalytic DRM and thermocatalytic DRM.

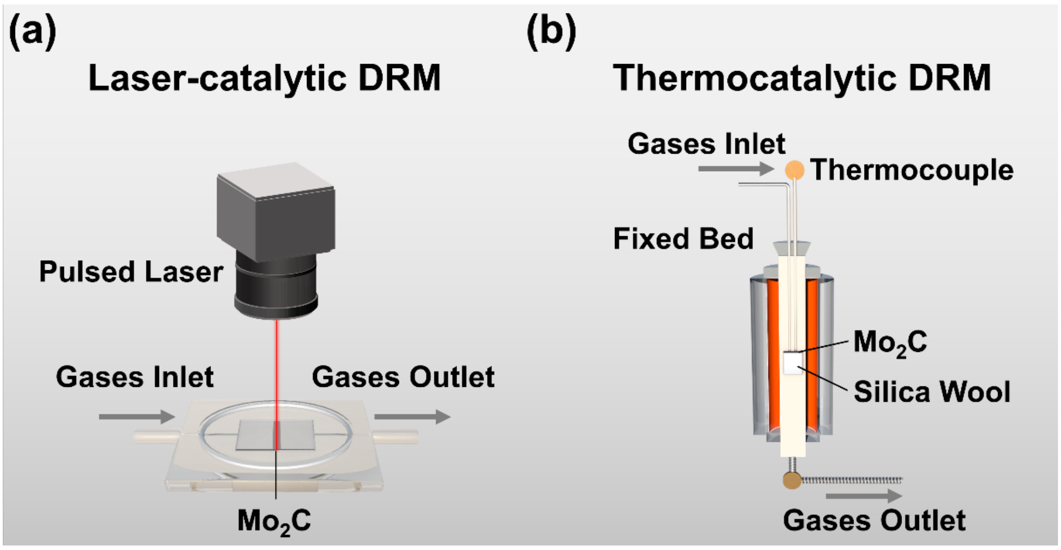

Supplementary Figure 27. Schematic diagram of (a) laser-catalytic DRM and (b) thermocatalytic DRM in flow systems.

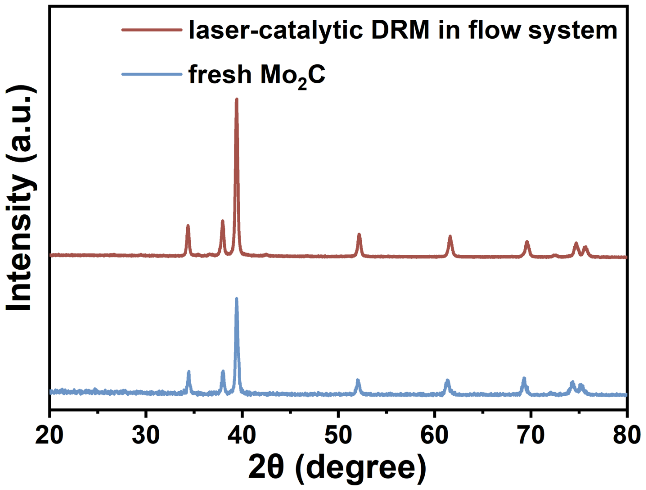

Supplementary Figure 28. XRD pattern of the catalyst after laser catalytic DRM in flow system for 160 mins.

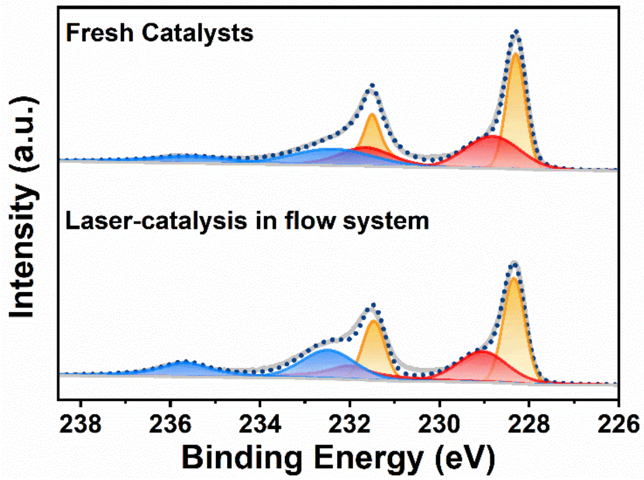

Supplementary Figure 29. Mo 3*d* XPS spectra of Mo_2C before and after laser-catalytic DRM in flow system for 160 mins.

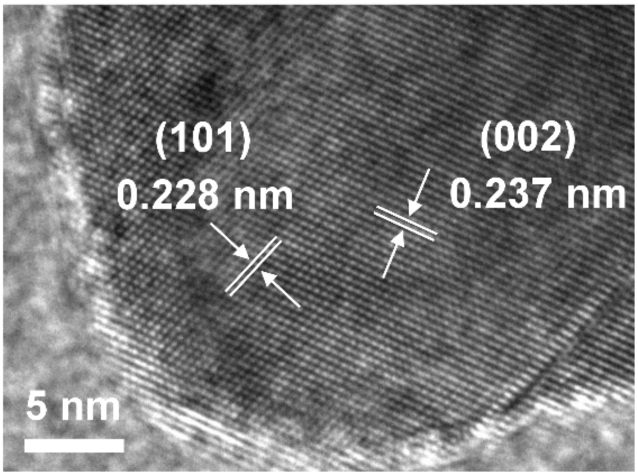

Supplementary Figure 30. HRTEM image of the catalyst after laser catalytic DRM in flow system for 160 mins.

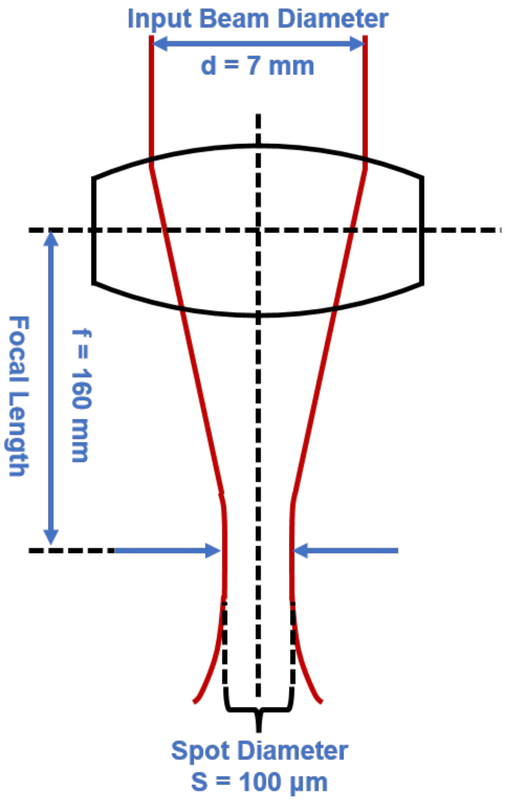

Supplementary Figure 31. Schematic diagram of laser beam focusing.

$$E=\frac{P_{avg}}{f_{rep}}=\frac{16\ \mathrm{W}}{20\ \mathrm{kHz}}=0.8\ \mathrm{mJ} \tag{14}$$

$$P_{peak}=\frac{P_{avg}}{f_{rep}\cdot\tau}=\frac{16\ W}{20\ kHz\cdot 100\ ns}=8\ kW \quad (15)$$

$$\text{Single pulse energy density}=\frac{E}{S}\ (mJ\ cm^{-2}) \quad (16)$$

Where E is the energy of a single pulse, P_{avg} is the average output power of the laser, f_{rep} is the repetition frequency, τ is the pulse width, P_{peak} is the peak power, and S is spot area.

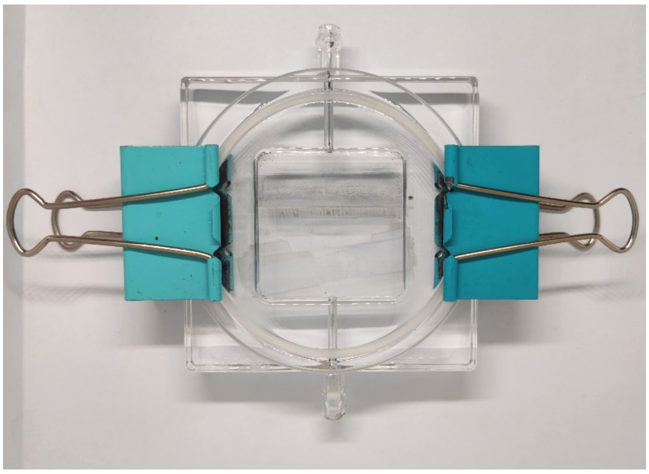

Supplementary Figure 32. Photograph of the reactor for laser-catalytic DRM in flow system.

Supplementary Tables

Table S1. Catalytic performance of the samples calculated from total Mo_2C mass within tablet area.

Sample	Tablet Area (mm^2)	Total Mo_2C Mass (mg)	Product rate (mmol/h)		Activity (mmol/h/g)	
			H_2	CO	H_2	CO
1	12.57	1.91	7.091	7.611	3712.0	3984.3
2	7.07	1.07	7.330	7.080	6850.5	6616.8
3	3.14	0.48	6.864	7.176	14300.8	14949.9
4	0.79	0.12	4.201	4.802	35000.0	40000.0

Table S2. Catalytic performance of the samples calculated from effective Mo_2C mass within hyperthermal area.

Sample	Thermal Area (mm^2)	Effective Mo_2C Mass (mg)[a]	Product rate (mmol/h)		Activity (mmol/h/g)	
			H_2	CO	H_2	CO
1	0.95	0.14	7.091	7.611	50642.9	54357.1
2	0.95	0.14	7.330	7.080	52357.1	50571.4
3	0.95	0.14	6.864	7.176	49000.0	51214.3
4	0.79	0.12	4.201	4.802	35000.0	40000.0

a. Effective Mo_2C Mass is defined as the amount of Mo_2C covered within a Thermal area of 0.95 mm^2.

Table S3. Catalytic performance of the samples calculated from effective Mo_2C mass within laser irradiation area.

Sample	Irradiated Area (mm^2)	Effective Mo_2C Mass (mg)[b]	Product rate (mmol/h)		Activity (mmol/h/g)	
			H_2	CO	H_2	CO
1	0.69	0.10	7.091	7.611	68173.1	73173.1
2	0.69	0.10	7.330	7.080	70480.8	68076.9
3	0.69	0.10	6.864	7.176	65961.5	68942.3
4	0.69	0.10	4.201	4.802	40384.6	46153.9

b. Effective Mo_2C Mass is defined as the amount of Mo_2C covered within a laser irradiation area of 0.69 mm^2.

Supplementary Table 4 Performance of some catalysts from recent studies.

Catalyst	Reaction Conditions	Catalyst mass (mg)	Production Rate (mmol h^{-1})		Yield (mmol h^{-1} g_{cat}^{-1})		H_2/CO	Ref
			H_2	CO	H_2	CO		
Mo_2C	Light Source:1064 nm Fiber laser (operating at 16 W); P = 1 atm; No additional thermal input	0.48	6.864	7.176	14300.8	14949.9	0.98	This work
Ni@HZSM-5	Thermocatalysis (CO_2:CH_4=3.1:1); P = 1 atm; T = 550 ºC	200 (Ni 0.85 wt.%)	2.866	5.275	1686 mmol h^{-1} g_{Ni}^{-1}	3103 mmol h^{-1} g_{Ni}^{-1}	-	3
Ni-Mo-MgO	Thermocatalysis; P = 1 atm; T= 800 ºC	50	93.750	107.100	1875	2142	0.88	4
Cu–Ru single-atom alloy	Light Source: white light from a supercontinuum laser (19.2 W cm^{-2}); P = 1 atm; No additional thermal input	1.5	2.279	2.295	1519	1530	0.99	5
SCM-Ni/SiO_2	Light Source: 500 W Xe lamp; P = 1 atm; No additional thermal input	24.3	24.932	29.014	1026	1194	0.86	6
$La_{0.9}Ca_{0.1}Fe_xNi_{1-x}O_{3-\delta}$	Thermocatalysis; P = 1 atm; T = 850 ºC	200	98.000	102.800	490	514	0.95	7
Ni-Fe Nanoalloy	Light Source: 300 W xenon lamp (3.62 W cm^{-2}); P = 0.18 MPa; No additional thermal input	5	1.600	3.100	320	620	0.52	8
Rh/CexWO3	Light Source: Xe lamp (300-1000 nm) 1.8 W·cm^{-2}; P = 1 atm; No additional thermal input	50 (no Rh wt.%)	-	-	88.5 mmol h^{-1} g_{Rh}^{-1}	152.3 mmol h^{-1} g_{Rh}^{-1}	0.59	9

Pt/TaN	Light Source: LA-251Xe lamp with L42 + HA30 filters (500–600 nm; 0.42 W cm^{-2}); P = 1 atm; T = 500 ºC	100	6.600	7.200	66	72	0.92	10
Rh/$SrTiO_3$	Light Source: 150 W Hg–Xe lamp; P = 1 atm; No additional thermal input	5	0.270	0.275	54	55	0.98	11
Ni/(PSC) CeO_2	Thermocatalysis; P = 1 atm; T = 450 ºC	-	-	-	32	48	0.67	12

Supplementary Table 5 Spot area and single pulse energy density of laser at different defocusing amount.

Defocusing Amount (mm)	Spot Area (mm^2)	Single pulse energy density (mJ/cm^2)
0	0.008	10000
5	0.080	1000
10	0.221	362
15	0.442	181
20	0.739	108
25	1.111	72

Supplementary Table 6 Laser-induced temperature under different laser irradiation conditions.

Laser irradiation conditions	Temperature (°C)	
	infrared thermal imager	tungsten-rhenium thermocouple
20 W pulsed laser; infocus	784	760
16 W pulsed laser; infocus	771	759
12 W pulsed laser; infocus	619	599
8 W pulsed laser; infocus	550	532
4 W pulsed laser; infocus	542	519
16 W; underfocus (DA = 5 mm)	753	742
16 W; underfocus (DA = 10 mm)	746	751
16 W; underfocus (DA = 15 mm)	775	789
16 W; underfocus (DA = 20 mm)	793	787
CW laser	789	791

Supplementary References

1. Wang, H. et al. H_2 Production from Methane Reforming over Molybdenum Carbide Catalysts: From Surface Properties and Reaction Mechanism to Catalyst Development. *ACS Catal.* **12**, 15501-15528 (2022).
2. A. Kurlov, E.B. Deeva, P.M. Abdala, D. Lebedev, A. Tsoukalou, A. Comas-Vives, A. Fedorov, C.R. Müller, Exploiting two-dimensional morphology of molybdenum oxycarbide to enable efficient catalytic dry reforming of methane, *Nat. Commun.* **11** (2020) 4920.
3. Zhu, Q. Y. et al. Enhanced CO_2 utilization in dry reforming of methane achieved through nickel-mediated hydrogen spillover in zeolite crystals. *Nat. Catal.* **5**, 1030–1037 (2022).
4. Song, Y. et al. Dry reforming of methane by stable Ni-Mo nanocatalysts on single-crystalline MgO. *Science* **367**, 777-781 (2020).
5. Zhou, L.A. et al. Light-driven methane dry reforming with single atomic site antenna-reactor plasmonic photocatalysts. *Nat. Energy* **5**, 61-70 (2020).
6. Huang, H. et al. Solar-light-driven CO_2 reduction by CH_4 on silica-cluster-modified Ni nanocrystals with a high solar-to-fuel efficiency and excellent durability. *Adv. Energy Mater.* **8**, 1702472 (2018).
7. Oh, J. et al. Precise modulation of triple-phase boundaries towards a highly functional exsolved catalyst for dry reforming of methane under a dilution-free system. *Angew. Chem. Int. Ed.* **61**, e202204990 (2022).
8. Zhao, J. Q. et al. NiFe nanoalloys derived from layered double hydroxides for photothermal synergistic reforming of CH_4 with CO_2. *Adv. Funct. Mater.* **32**, 2204056 (2022).
9. Yang, Y. Y. et al. Light-induced redox looping of a rhodium/Ce_xWO_3 photocatalyst for highly active and robust dry reforming of methane. *Angew. Chem. Int. Ed.* **61** (2022).
10. Liu, H. M., Song, H., Zhou, W., Meng, X. G. & Ye, J. H. A Promising application of optical hexagonal TaN in photocatalytic reactions. *Angew. Chem. Int. Ed.* **57**, 16781-16784 (2018).

11. Shoji, S. et al. Photocatalytic uphill conversion of natural gas beyond the limitation of thermal reaction systems. *Nat. Catal.***3**, 148–153 (2020).

12. Xiao, Y. C. & Xie, K. Active exsolved metal-oxide interfaces in porous single-crystalline ceria monoliths for efficient and durable CH_4/CO_2 reforming. *Angew. Chem. Int. Ed.* **61** (2022).
